# A 3D reconstruction based on an unsupervised domain adaptive for binocular endoscopy

**DOI:** 10.3389/fphys.2022.994343

**Published:** 2022-09-01

**Authors:** Guo Zhang, Zhiwei Huang, Jinzhao Lin, Zhangyong Li, Enling Cao, Yu Pang, Weiwei sun

**Affiliations:** ^1^ School of Communication and Information Engineering, Chongqing University of Posts and Telecommunication, Chongqing, China; ^2^ School of Medical Information and Engineering, Southwest Medical University, Luzhou, China; ^3^ School of Optoelectronic Engineering, Chongqing University of Posts and Telecommunication, Chongqing, China; ^4^ School of Bioinformatics, Chongqing University of Posts and Telecommunication, Chongqing, China; ^5^ School of Software Engineering, Chongqing University of Posts and Telecommunication, Chongqing, China

**Keywords:** adaptive, deep learning, binocular endoscopic, smoke, three-dimensional

## Abstract

In minimally invasive surgery, endoscopic image quality plays a crucial role in surgery. Aiming at the lack of a real parallax in binocular endoscopic images, this article proposes an unsupervised adaptive neural network. The network combines adaptive smoke removal, depth estimation of binocular endoscopic images, and the 3D display of high-quality endoscopic images. We simulated the smoke generated during surgery by artificially adding fog. The training images of U-Net fused by Laplacian pyramid are introduced to improve the network’s ability to extract intermediate features. We introduce Convolutional Block Attention Module to obtain the optimal parameters of each layer of the network. We utilized the disparity transformation relationship between left- and right-eye images to combine the left-eye images with disparity in HS-Resnet to obtain virtual right-eye images as labels for self-supervised training. This method extracts and fuses the parallax images at different scale levels of the decoder, making the generated parallax images more complete and smoother. A large number of experimental research results show that the scheme can remove the smoke generated during the operation, effectively reconstruct the 3D image of the tissue structure of the binocular endoscope, and at the same time, preserve the contour, edge, detail, and texture of the blood vessels in the medical image. Compared with the existing similar schemes, various indicators have been greatly improved. It has good clinical application prospects.

## 1 Introduction

With the development of society, image processing (Li et al., 2016a; Li et al. 2016b; Li et al. 2018) is widely used in the medical field. During clinical surgery, the quality of medical images is degraded by noise. Noise is mainly composed of blood, light changes, specular reflection, smoke, etc. Among them, the smoke generated by laser and electrocautery-based human tissue ablations will significantly reduce the imaging quality of the lesion area. The results will affect the doctor’s judgment, prolong the operation time, and increase the operation risk. Therefore, it is necessary to remove the smoke by physical means and purify it by image-processing algorithms (Kotwal et al., 2016; Yang and Sun, 2018; Chen et al., 2019a; Sidorov et al., 2020; Venkatesh et al., 2020). In addition, the particularity of the human tissue and imaging conditions are limited. Due to the influence of equipment light source and thermal noise acquisition, the quality of the collected endoscopic images is generally not high. Images obtained directly by endoscopy tend to have low imaging quality, resulting in the loss of some vascular tissue characteristics. Therefore, for the accuracy and convenience of later diagnosis, it is particularly important to improve the recognition ability of endoscopic images, filter out noise, and enhance the outline of the vascular tissue by reconstructing 3D details.

In the 3D display research based on the traditional stereo-matching method, the pixels of the left- and right-eye images have a parallax correspondence, and the 3D display can be performed after the parallax is obtained from the algorithm model (Hu et al., 2012; Besse et al., 2014; Yang and Liu, 2014; Penza et al., 2016). Compared with traditional algorithms, the method based on visual Simultaneous Localization and Mapping (SLAM) is slightly better in real-time performance. Most SLAM algorithms perform an inter-frame estimation and loop closure detection through feature point-matching techniques. Although the SLAM-based method only regards depth estimation as an intermediate product, its double-end depth estimation network provides a clear idea for subsequent research. Many subsequent articles have used its basic model (Mahmoud et al., 2016; Yi et al., 2016; Vijayanarasimhan et al., 2017; Wang et al., 2018a; Qiu and Ren, 2020). However, for the complex tissues and organs of the human body, traditional methods cannot meet the requirements of medical scenarios in terms of 3D reconstruction time and accuracy. In the research of 3D displays based on the neural network, researchers conducted supervised training on natural scene datasets containing depth labels. The final test can achieve the effect of real-time depth estimation (Antal, 2016; Kendall et al., 2017; Pang et al., 2017; Huang et al., 2018; Luo et al., 2019; Zhang et al., 2019). Since medical endoscopic images contain fewer datasets with depth labels (Penza et al., 2018), unsupervised learning is more suitable for 3D display of binocular laparoscopic images (Shurrab and Duwairi, 2022). A novel self-supervised learning strategy based on context restoration in order to better exploit unlabeled images (Chen et al., 2019b; Chen et al., 2022). The virtual viewpoint is obtained as a label through an implicit function, and the neural network is calculated and solved. Researchers can avoid a lot of dataset labeling work (Garg et al., 2016; Feng et al., 2017; Kendall et al., 2017; Zhou et al., 2017; Yin and Shi, 2018; Wang et al., 2019a; Tosi et al., 2019; Taleb et al., 2021).

**FIGURE 1:**
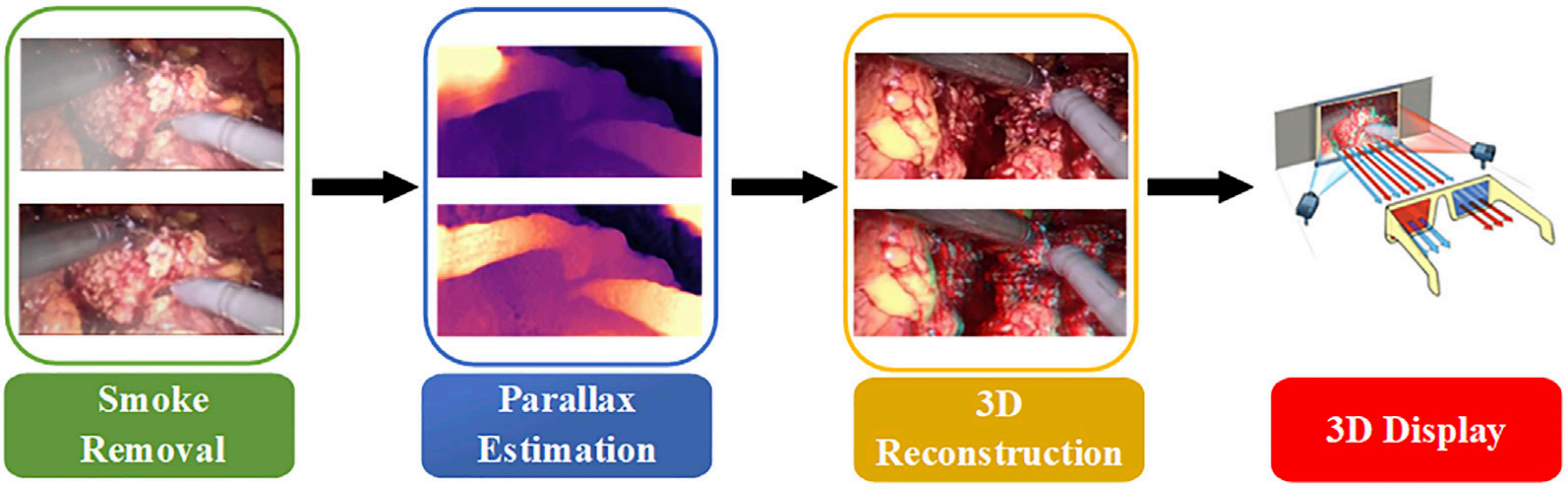
Overall process flow chart.

In fact, it is often necessary to preprocess the image to remove various noises in the application of traditional methods and neural network schemes. Although the performance of neural networks on endoscopic images increases with the number of neurons, the complexity of convolution operations is very high. This leads to a blind increase in the size of the network and consumes a lot of training time. Therefore, combined with the real-time application requirements of clinical operations and the imaging characteristics of binocular endoscopes, we propose a 3D reconstruction method of binocular endoscope medical images based on adaptive neural network. The overall flow chart of the process is shown in Figure 1. The main contributions of this paper can be summarized as follows:

1) We proposed an improved U-NET adaptive network model for the smoke generated during laparoscopic surgery. We added training images fused by Laplacian pyramids at each layer of the encoder. A lightweight Convolutional Block Attention Module (CBAM) (Woo et al., 2018) attention mechanism module was added to the last five layers of the decoder to improve the network’s ability to extract intermediate features. The processing time of a single image reaches 90.19 pfs, which can purify endoscopic surgical smoke in real time.
2) In view of the lack of true parallax in binocular endoscope images, we propose an improved HS-Resnet network. The left-eye image is combined with disparity to obtain a virtual right-eye image as a label for self-supervised training. In the process of feature extraction, multi-scale segmentation and synthesis are performed so that the network can effectively extract different scale features of various receptive fields. We reconstructed 3D structures with visibility and realism.
3) We proposed a color-difference 3D reconstruction scheme which separates the red component of the original image and combines the parallax, and fuses the combined red component with the blue–green component of the original image to obtain a 3D image. This can effectively reduce the details and color loss of the endoscopic image and retain the details of the medical images.

## 2 Methods

### 2.1 Smoke removal

#### 2.1.1 Smoke synthesis

**FIGURE 2:**
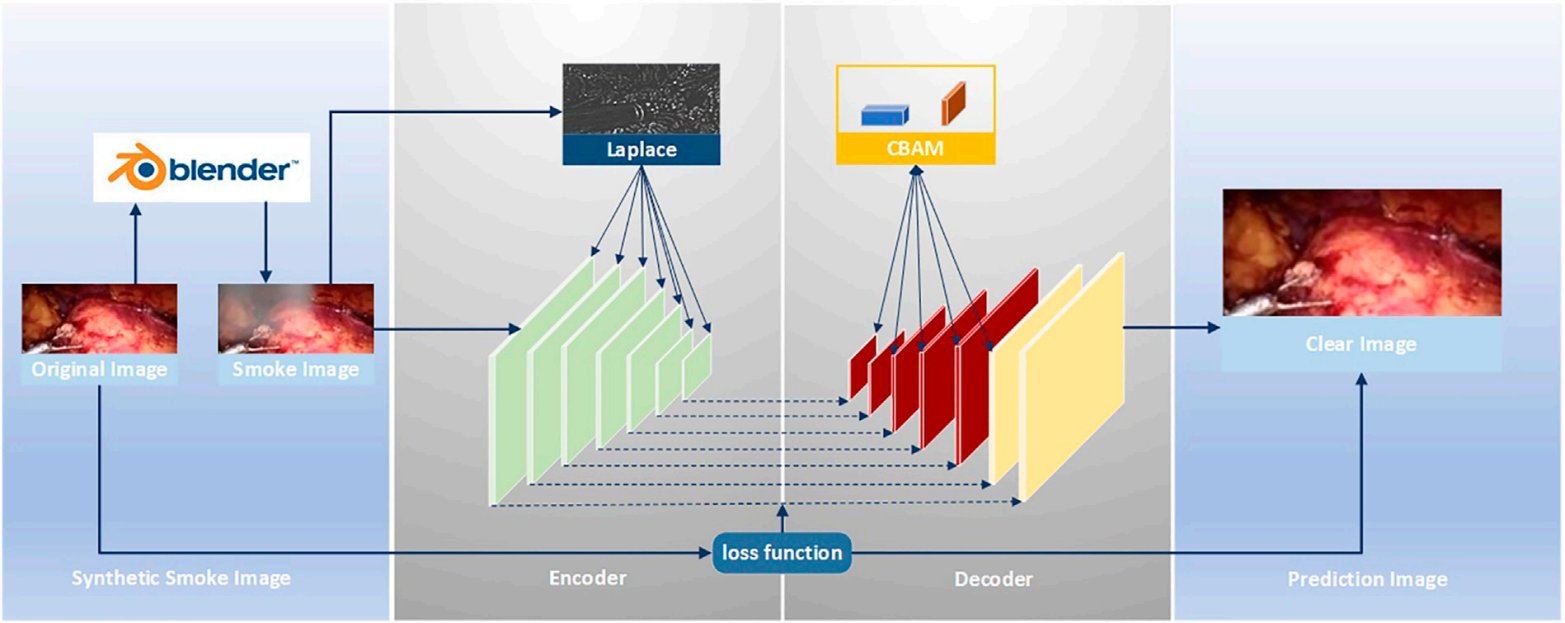
Smoke removal flow chart.

The improved U-Net (Zhou et al., 2021) model is used to realize the smoke purification function of endoscopic images. The steps of the smoke purification model are shown in Figure 2 below. Due to the lack of medical endoscopic image datasets containing real labels, we used the Render software to add smoke to real laparoscopy images as training images, and used the original images without smoke as labels. The loss function was obtained by comparing the purified image obtained by the model and the label, and back propagation reduced the loss to obtain the parameters of each layer of the network. In the network design, in order to increase the network’s ability to retain image details and colors, we added the Rapp to the encoder. For the original fog image of Laplace fusion, the scale of Laplace transform is the same as that of the encoder. In order to improve the network’s performance, we added the CBAM attention mechanism to the last five layers of the decoder to use the synthetic image containing smoke as the training set. The original image is sent to the improved U-Net model as a training set label for training. Through back propagation, each layer of the network obtains the corresponding parameters. Finally, the test set is sent to the model to predict the purified image.

The medical scene dataset in the field of smoke purification is relatively rare. There is currently no dataset containing real labels. Therefore, either unsupervised learning can be used or software can be used to add smoke to medical endoscopic images. Then, use the images without the added smoke as labels. In the two schemes, simple supervised learning can be used to solve the problem after smoke synthesis, and supervised learning is mature in the field of deep neural networks. So, we choose the scheme of artificially synthesizing smoke. The smoke generated in laparoscopic surgery is usually generated randomly and has nothing to do with the depth. The modern image-rendering engines have a complete built-in model. This can better simulate the shape of the smoke compared to physical solutions. Therefore, we used the 3D graphics-rendering engine. Render to the training images are obtained by rendering the smoke on laparoscopic images that do not contain smoke.

The smoke is rendered by the rendering engine and has local color and transparency. The smoke is controlled by the input parameters Trad, Drand, and Position, as shown in formula (1):

$$I_{\mathrm{smoke}\left(x,y\right)}=\mathrm{Blender}\left(T_{\mathrm{rand}},D_{\mathrm{rand}},P_{\mathrm{rand}}\right)$$ (1)

Using Render to fog the laparoscopic image, the rendered smoke is similar to the real smoke. It has the characteristics of local pure white and transparency. The fogged image is superimposed by the original image and random smoke, as shown in formula (2):

$$I_{s-\mathrm{image}}\left(x,y\right)=I_{s-\mathrm{free}}\left(x,y\right)+I_{\mathrm{smoke}}$$ (2)

The smoke added to the laparoscope is obtained by superimposing the luminance values of the rendered R, G, and B channels proportionally. The ratio is shown in formula (3):

$$I_{\mathrm{mask}}\left(x,y\right)=\left(0.3^{*}I_{\mathrm{smoke}}{\left(x,y\right)}^{R}\right)+\left(0.59^{*}I_{\mathrm{smoke}}{\left(x,y\right)}^{G}\right)+\left(0.11^{*}I_{\mathrm{smoke}}{\left(x,y\right)}^{B}\right)$$ (3)

To better simulate light smoke, fog, and thick smoke fog, we rendered two types of fog. Firstly, images without fog are selected as the original training set in the dataset. In rendering, the original dataset is randomly added fog using the data settings of the aforementioned formula. We added primary smoke as the light fog dataset. Then, the light fog dataset is sent into the rendering for secondary random adding fog to obtain the thick fog dataset. Finally, training is performed on the thick fog dataset and the light fog dataset, respectively.

#### 2.1.2 Improved U-net network

**FIGURE 3:**
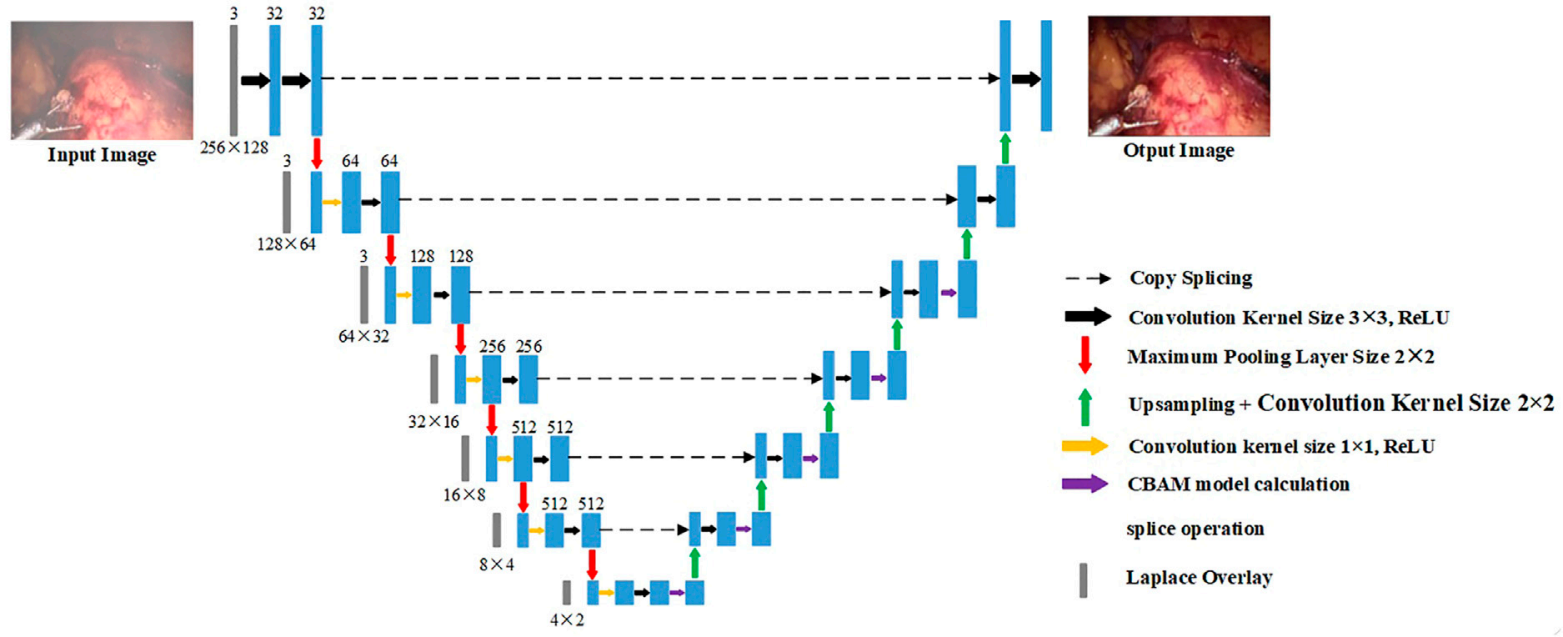
Encoder–decoder network.

For the original U-Net, it is found through experiments that it cannot effectively purify the smoke, or the image resolution decreases after purifying the smoke. This is due to the loss of image details in the process of up-sampling and down-sampling. But for medical scenes, the loss of detail information will seriously affect the doctor’s judgment. Therefore, we added the training image fused by the Laplacian pyramid in the down-sampling part to compensate for the loss of details of the image during the down-sampling process. The image fusion of the Laplacian image pyramid is equivalent to a filter, which maps the image to different frequency bands. The features are learned, and fusion operations are performed on each frequency band, thereby effectively preserving image details on each frequency band. The U-Net model is improved according to the characteristics of medical endoscopy, as shown in Figure 3.

The down-sampling part on the left of Figure 3 is the encoder. The encoder can extract features of different scales of endoscopic images through convolution operations. Each layer of the encoder corresponds to splicing, two convolutions, and a max pooling operation. Laplace the superposition operation refers to the fusion of the Laplacian pyramid image for the input training image according to the size of each layer of the encoder. The transformed image and the features of each layer are spliced and sent to training. The seven convolution groups are named conv1 to conv7, respectively. The size of the convolution kernel of each layer is 7 × 7, 5 × 5, 3 × 3, 3 × 3, 3 × 3, 3 × 3, and 3 × 3. Each layer is convolved twice. The strides of the two convolutions are 1 and 2, respectively. The number of output layers per layer is 32, 64, 128, 256, 512, 512, and 512. Therefore, the encoder down-sampling factor is 64.

The decoder restores the down-sampled image to its original size. The CBAM attention module is inserted into the first five layers of the decoding part of the U-Net network, as shown in the up-sampling part on the right side of Figure 3. The decoder also adopts 7 sets of convolutions; each group contains two up-sampling layers with steps 1 and 2. The size of the convolution kernel is all 3 × 3, and the number of output layers is 512, 512, 256, 128, 64, 32, and 16, respectively. In addition, there are corresponding connections between the encoders and decoders where the features of the lower layers are connected with the features of higher layers. Information from the higher layers can be directly transmitted to the bottom layer of the network to prevent the loss of high-quality details.

The loss function of the improved U-Net network is the minimum absolute value deviation loss of the original image and the synthetic smoke image, as shown in formula (4):

$$L=\sum_{xy}\left|I_{\mathrm{original}}\left(x,y\right)-I_{\mathrm{desmoked}}\left(x,y\right)\right|$$ (4)

#### 2.1.3 Laplacian image pyramid fusion

The maximum pooling operation is used in the down-sampling process. Due to the continuous down-sampling operation, the image details are lost in each frequency domain. In order to better preserve the image quality in the specified frequency domain, a Laplacian image is introduced in the encoder part of the pyramid fusion. This method uses the nearest point interpolation when up-sampling the image after Gaussian sampling. Especially in the place where the image gradient changes greatly, the problem of sudden change of the pixel value occurs easily. The image details are lost, and there may be mosaic or sawtooth noise (Wang et al., 2019b). This article uses bidirectional interpolation to replace the nearest neighbor interpolation to improve this problem. It processes the four direct neighbors near the sample point. The image quality is higher after processing.

The Laplacian-transformed smoke image is added before each convolutional layer in the encoder, and the main process of the Laplacian pyramid fusion is shown in formula 5:

$$L_{i}\left(I\right)=G_{i}\left(I\right)-\mathrm{up}\left(\mathrm{down}\left(G_{i}\left(I\right)\right)\right)$$ (5)

where I represents the original image containing smoke; i represents the level pyramid. $\mathrm{up}\left(\mathrm{down}\left(G_{i}\left(I\right)\right)\right)$ represents the up-sampled lower-layer Gaussian sampled image; and $G_{i}\left(I\right)$ represents the Gaussian sampled image.

**FIGURE 4:**
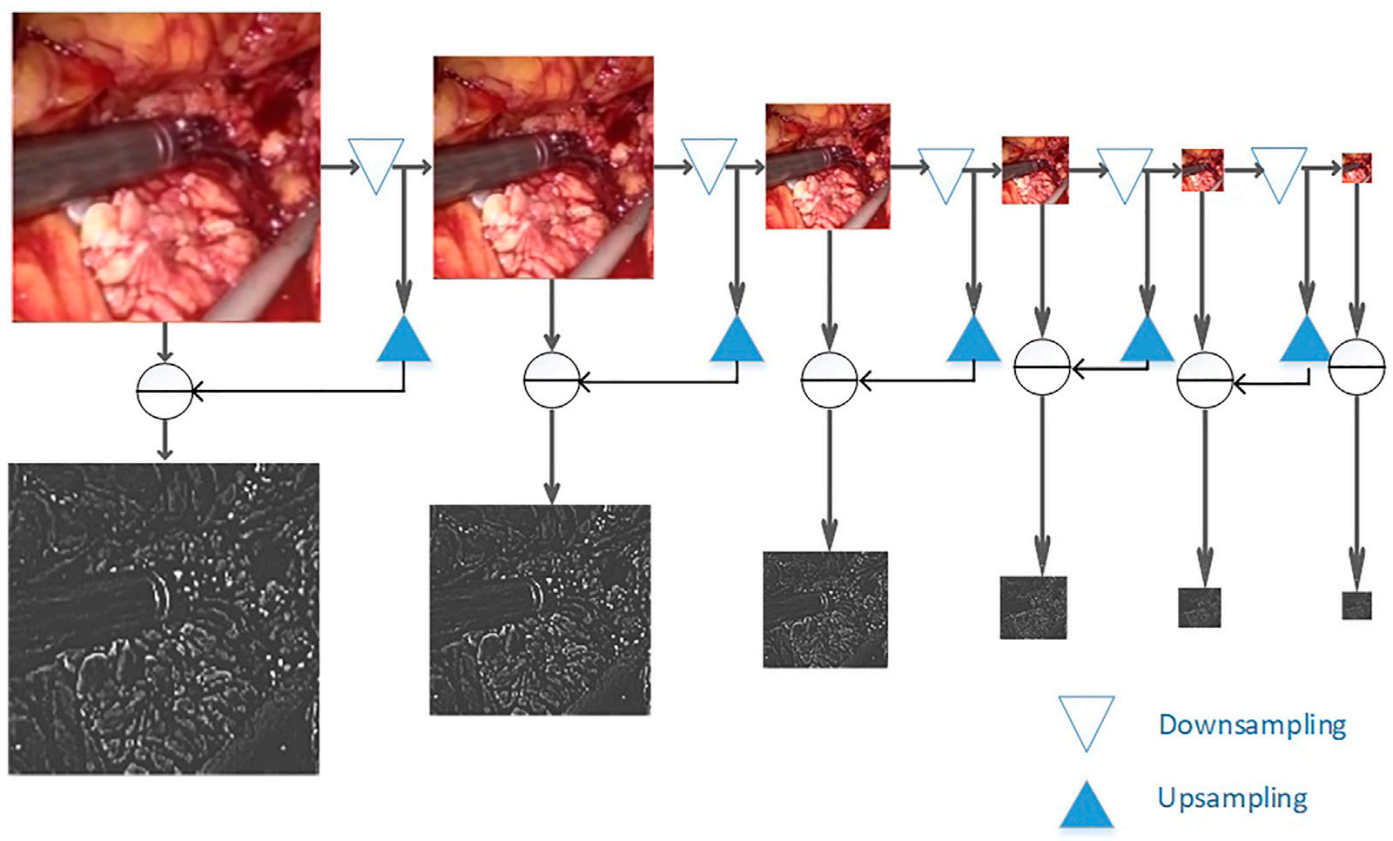
Laplacian image pyramid decomposition.

As shown in Figure 4, to smoothly image the image to different frequency bands, we performed Gaussian down-sampling on the endoscopic image. As shown in the color endoscopic image, as the number of Gaussian sampling increases, the size of the endoscopic image becomes smaller. But it can retain the more important pieces of information in the image. For a Laplacian-transformed image of a specific size, Gaussian down-sampling is performed according to the specified scale, and then the Laplacian pyramid fusion image is obtained. As shown in the black and white image, the Laplacian pyramid fusion image (in order to make the image easy to observe, the brightness value of the Laplacian fusion image is increased) effectively retains the line and edge information of the image. The size is the same as the U-Net down-sampling size. Therefore, it can be directly superimposed and spliced with the input feature image in the network and then be sent to the network for training. Finally, this article splices it to the corresponding size of the convolutional layer to participate in training.

#### 2.1.4 CBAM attention mechanism

The CBAM attention mechanism module is lightweight and effective. Therefore, we inserted the CBAM attention mechanism module in the last five layers of the decoder; following the network to participate in the training process to improve the feature selection performance of the model. For any input feature, CBAM obtains attention features along two independent dimensions of channel and space. The original input feature is optimized by multiplying the attention feature with the original input feature image. The specific process is as follows: for the input feature image $F\in R^{C*H*W}$ of any size, CBAM will calculate a one-dimensional channel feature image $M_{c}\in R^{C*1*1}$ . A two-dimensional spatial feature image $M_{c}\in R^{1*H*W}$ is derived in the blue part of Supplementary Figure S1. The orange part is shown in formulas 6 and 7. The blue part of the channel information and the orange part of the spatial information are fused with the original input feature to obtain the optimized input feature. This feature is used as the next input to the convolutional layer.

$$F^{\prime }=M_{c}\left(F\right)⊗F$$ (6)

$$F^{\prime \prime }=M_{s}\left(F^{\prime }\right)⊗F^{\prime }$$ (7)

We treat each channel of input features as a feature detector, as shown in the blue part of Supplementary Figure S1. Channel attention is used to pay attention to the content of the input image, so the features are compressed into a “pipe”. Observe the image content that still exists after the reduction, and find its calculation method as follows: First, average the pooling and summing of the input features according to their feature-stacking direction. Convolution is performed after max pooling, followed by the activation of the result of the convolution, and finally the feature fusion. As shown in formulas 8 and 9:

$$M_{c}\left(F\right)=\sigma \left(MLP\left(AvgPool\left(F\right)\right)+MLP\left(MaxPool\left(F\right)\right)\right)$$ (8)

$$M_{c}\left(F\right)=\sigma \left(W_{1}\left(W_{0}\left(F_{avg}^{c}\right)\right)+W_{1}\left(W_{0}\left(F_{\max}^{c}\right)\right)\right)$$ (9)

where $W_{0}\in R^{C/r*C}$ and $W_{1}\in R^{C*C/r}$ , using ReLU as the activation function after $W_{0}$ .

Channel attention pays attention to the key positions of the image. Spatial attention compresses the feature dimension into an “image”, which is convenient for the neural network to identify the position of the image object. As shown in the orange part of Supplementary Figure S1, two different feature descriptions $F_{max}^{s}\in R_{1*H*W}$ and $F_{avg}^{s}\in R_{1*H*W}$ are obtained by using max pooling and average pooling in the dimension of the channel, and then, the aggregation operation is used to generate the spatial feature image $M_{s}\left(F\right)\in R_{H\text{*}W}$ . As shown in formulas 10 and 11:

$$M_{s}=\sigma \left(f^{7*7}\left(\left[AvgPool\left(F\right);MaxPool\left(F\right)\right]\right)\right)$$ (10)

$$L=\sum_{xy}\left|I_{\mathrm{original}}\left(x,y\right)-I_{\mathrm{desmoked}}\left(x,y\right)\right|$$ (11)

### 2.2 A method for estimating binocular disparity in endoscope is proposed

**FIGURE 5:**
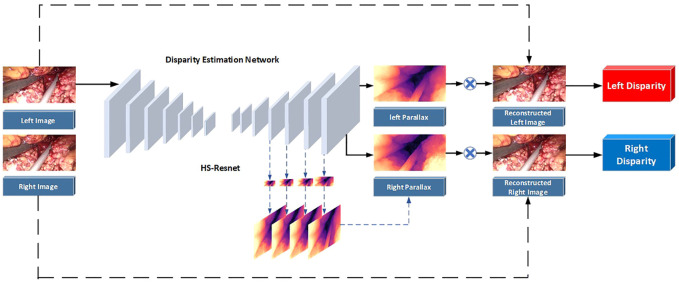
Flowchart of the parallax estimation algorithm.

The parallax estimation method of a binocular endoscopic image based on self-supervised deep learning is shown in Figure 5. The corrected left and right images are used as inputs. The left image is used as a standard input into a convolutional neural network for training. The left and right original images are used as labels to provide supervision information for the network.

Step 1 The corrected left and right images are taken as the training images, and the left image is sent into the neural network for training. Then, the initial left and right parallax images are obtained by a CNN non-linear function fitting.

Step 2 The left and right parallax images obtained from the network can be regarded as the deviation between the left and right views. So, the virtual right image can be obtained by combining the original left image and the left parallax through bilinear interpolation. The virtual left image can be obtained by combining the original right image and the right parallax.

Step 3 Reverse propagation is carried out by comparing the difference between the virtual left view and the real left view and between the virtual right view and the real right view. Appropriate parameters can be obtained for each layer of the network.

#### 2.2.1 CBAM attention mechanism

The encoder is used to construct the U-NET structure with ResNet as the convolutional layer of the network, extracting the features of endoscope images. The size of the images is restored to the original size through the decoder. Specifically, the encoder first preprocesses the convolution for the inputted RGB images, with a convolution kernel size of 7 × 7, step length of 2, and zero fill of 3. After preprocessing, the image is batch normalized, followed by 4 convolutions with a convolution kernel of 3 × 3. After 5 convolutions, the feature dimensions of the convolution kernel size are 16, 32, 64, 128, and 256.

**FIGURE 6:**
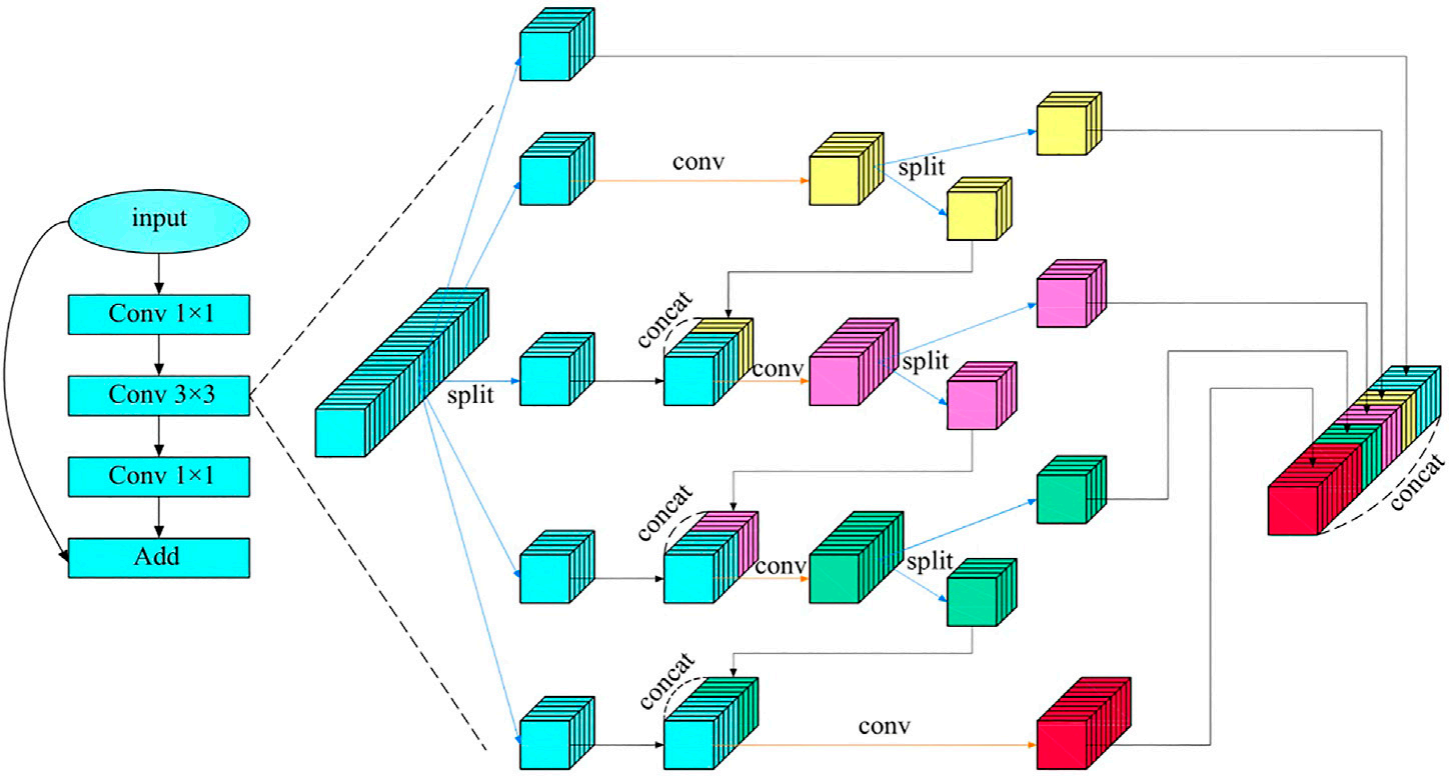
HSB module.

Multi-scale features are particularly important in machine vision, which can image features to multiple frequency domains and be conducive to keeping detailed features of images. Focusing on medical endoscope images that require highly detailed features, an HS-Resnet containing multi-scale features is adopted (Godard et al., 2017). It contains a hierarchical separation module embedded in the convolutional module of the deep network, where HSB can effectively improve the performance of the network and HS-ResNet 50 can achieve 81.28% of the datasets on ImageNet, exceeding the current optimal effect of ResNet. As shown in the Figure 6, HS-Resnet is composed of multiple segmentation and splicing operations, of which the hierarchical segmentation and splicing operations together constitute the HSB multi-scale feature extractor.

HSB contains two main operations: split and concatenate. Among these two, split is used for feature grouping and to make the two groups after grouping have the same number of channels. When the number of features to be grouped is odd and the channel number of the two groups after the split operation is different, one part can be regarded directly as the output, equivalent to identity imaging, and the other part can be used as the input to the next convolution layer for a more in-depth and detailed feature extraction. The concatenate operation fuses features with the same size but different contents so that features with different convolution degrees can carry out information interaction. When the concatenate operation adopts a simple superposition operation, the characterization ability of the original features can be better ensured.

Figure 6 shows the structure of HSB, where HS-Resnet uses a deep residual module and HSB processes features in the 3 × 3 convolution layer. The input features are divided into S groups $x_{i}$ with the same number of channels after 1 × 1 convolution. Then, after passing through a 3 × 3 convolution layer $F_{i}\left(\right)$ times, $x_{i}$ becomes $y_{i}$ , which can be divided into $y_{i,1}$ , $\;y_{i,2}$ , $y_{i,3}$ , $y_{i,4}$ , and $y_{i,5}$ . Among them, $y_{i,1}$ is added directly to the layer and to the output $x_{i+1}$ , similar to the green feature on the top. $y_{i,2}$ is segmented into two groups with yellow features after the convolution operation, where one group is added to the layer and to output $x_{i+1}$ . The other group is sent to the convolution layer after matching with $y_{i,3}$ . Similarly, $y_{i,3}$ is divided into two groups with red features, where one group is added to the layer and the output $x_{i+1}$ . The other group is sent to the convolution layer to obtain the green feature after matching with $y_{i,4}$ . $y_{i,4}$ is processed same as $y_{i,3}$ . Finally, the feature of $y_{i,5}$ after the convolution operation will be taken as the last part of the layer’s output. After such continuous processing, the features are equivalent to more scale and deeper convolution. The small receptive field in the final output feature can pay attention to the detail part and enhance the processing ability of the network for small features.

Figure 6 shows the situation in which s is set to 5. In fact, a larger number of groups can achieve the extraction performance of more scales. A larger number of channels means richer features requiring more parameters. Therefore, it is necessary to choose between the number of parameters and the capability of feature extraction.

$$y_{i}=\{\begin{array}{cc} x_{i} & i=1\\ f_{i}\left(x_{i}⊕y_{i-1,2}\right) & 1<i\leq s \end{array}$$ (12)

HSB does not increase the number of parameters in the network. Compared with a standard convolution, it even has fewer parameters. The standard parameter complexity is shown in Formula 13:

$$P_{normal}=k\times k\times s\times w\times s\times w=k^{2}\times s^{2}\times w^{2}$$ (13)

The complexity of HSB is shown in Formula 14:

$$P_{HSB}=\{\begin{array}{cc} 0, & i=1\\ k^{2}\times w^{2}\times \left(\frac{2^{s-1}-1}{2^{s-1}}+1\right) & 1<i\leq s \end{array}$$ (14)

It can be seen from the comparison between Formulas 13 and 15 that the complexity analysis of HSB is actually smaller than that of an ordinary convolution.

$$k^{2}\times w^{2}\times \left(\frac{2^{s-1}-1}{2^{s-1}}+1\right)\leq k^{2}\times w^{2}\times \left(\frac{2^{s-1}-1}{2^{s-1}}+s-1\right)<k^{2}\times w^{2}\times \left(s-1+s-1\right)=k^{2}\times w^{2}\times \left(2s-2\right)<k^{2}\times w^{2}\times s^{2}$$ (15)

#### 2.2.2 Multi-scale decoder

The decoder is the deconvolution process of the encoder, aiming to restore the image to the original image size. The decoder up-samples the image, which includes a 3 × 3 deconvolution, to restore each layer of the image to the same size as the decoder. The output feature dimensions of each convolution are 256, 128, 64, 32, and 16. Bilinear sampling has gradient locality, and may not converge to the global minimum during the training process of the final disparity estimation. Therefore, the disparity is extracted from the last four layers of the filter during decoding. And then, the disparity calculation loss function of each layer is fused into the final loss function solution. Each layer calculates the loss function according to different image sizes. Due to the severe compression of low-resolution images, it is difficult to retain important details of the image. Parallax discontinuities are prone to occur in the weak repeating parts of the tissue structure, because the photometric errors at these locations are blurred and inaccurate. Inspired by binocular stereo vision, we improved the loss function and reconstructed the disparity image in the last four layers of the decoder with different image sizes. The loss functions of different scales were calculated.

#### 2.2.3 Improved loss function

1) Photometric reconstruction loss

Self-supervised learning mainly uses the disparity relationship between the left and right images of the binocular endoscopic image to establish a loss function (Godard et al., 2019). The training loss is expressed as a photometric re-projection loss, which is used to describe the difference between the virtual viewpoint and the real view. The total loss is obtained by adding the losses of all pixel points. The calculation process of the loss function is shown in formula 16:

$$L\left(p\right)=\sum pt\left(I_{t},I\prime_{t}\right)$$ (16)

$I_{t}$ is the original image, $I\prime_{t}$ is the virtual view, and pt is the difference between the two images. The total photometric loss is obtained by combining the differences of all images (Zhao et al., 2019). The structural similarity index SSIM is used to characterize the photometric reconstruction error. The specific calculation process is shown in formula 17:

$$pt\left(I_{t},I\prime_{t}\right)=\frac{\alpha }{2}\left(1-SSIM\left(I_{t},I\prime_{t}\right)\right)+\left(1-\alpha \right){\left\|I_{t},I\prime_{t}\right\|}_{1}$$ (17)

Among them, α is the weight coefficient between SSIM and L1 norms, which can be obtained from training experience. We set it as α = 0.85. During training, the model extracts image features from the left image in the binocular laparoscopic image to obtain the initial disparity. Then, it use the left image and the original image to linearly translate to get the virtual right image, and then compare the real right image with the original right image to get the loss. Image sampling is performed using Spatial Transformer Networks (STN) (Jaderberg et al., 2015). The original image is sampled with the disparity image as the standard, and the STN takes the weighted sum of the surrounding four pixels for each sampling point. Its calculation process can be differentiated and can follow the neural network to participate in the process of back propagation.

As shown in formula 16, the existing literature generally averages the re-projection loss across all training images when calculating the photometric reconstruction error for self-supervised depth estimation. This has some problems in consecutive images. Certain matching feature points do not match in the occluded image. This leads to a large error in photometric reconstruction. However, the loss function is averaged, so that the two points cannot be correctly matched. Then, the obtained disparity image or depth image is blurred. Pixels that are easily occluded during continuous motion mainly come from the boundaries of moving objects. For example, in the process of laparoscopic surgery, the forceps move more frequently and there will be a long-term or short-term occlusion in the patient’s body. The background in the human body cannot be matched. For the photometric reconstruction loss of the same pixel appearing in different images, this article adopts the minimum value instead of the average value to improve the photometric loss. As shown in formula 18:

$$L_{p}=\sum_{t\prime }pe\left(I_{t},I_{t\prime \to t}\right)\Rightarrow L_{p}=\underset{t\prime }{\min}pe\left(I_{t},I_{t\prime \to t}\right)$$ (18)

For all pixels in an image, it is not necessary to calculate the loss function in its entirety. We use an automatic masking scheme that preserves points that move relative to the camera and removes points that are stationary relative to the camera. For example, in laparoscopic surgery, when the abdominal lens is rotated, all pixels move with the lens. At this point, all pixel point losses are calculated. When the abdominal lens remains stationary, the background of the internal abdominal cavity that the endoscope can look into is fixed. As the forceps moves the abdominal tissue relative to the lens, only the moving portion is counted when calculating the loss. The rest of the points are removed, and the removed part is called a mask. The mask is computed by the network. Masked pixels can be characterized as a static camera, which is equivalent to being relatively stationary with the camera, or can represent low-texture areas.

This article uses the binary mask parameter μ∈{0,1} . Among all loss functions, μ is only related to the photometric reconstruction loss, as shown in formula 19:

$$\mu =\left[\underset{t\prime }{\min}pe\left(I_{t},I_{t\prime \to t}\right)<\underset{t\prime }{\min}pe\left(I_{t},I\prime_{t}\right)\right]$$ (19)

2) Left–right consistency loss

Our proposed photometric reconstruction error can examine the similarity between the original view and the virtual view. The left and right consistency loss is used to measure the similarity between the left and right disparity images generated by the network. The disparity acquisition module only has the left image as input, but needs to predict the left and right binocular disparity images. Therefore, the similarity between the left and right disparity images needs to be constrained. A virtual right disparity image can be obtained by linearly transforming the left image disparity on each pixel using right image disparity. The original right disparity image is compared with the virtual right disparity image, and the L1 norm is obtained as the left–right consistency loss. The left and right consistency losses can constrain the left and right parallaxes to ensure the accuracy and continuity of the parallax. In order to reconstruct the loss obtained from the right disparity, we also calculated the loss to reconstruct the left disparity during training, as shown in formula 20:

$$L_{lr}^{l}=\frac{1}{N}\sum_{i,j}\left|d_{ij}^{l}-d_{ij+d_{ij}^{l}}^{r}\right|$$ (20)

3) Edge-smoothing loss

There is a very strong connection between adjacent disparity images. Constraining the transformation magnitude of disparity through a loss function can effectively improve the problem of discontinuous disparity. Parallax can also be locally smoothed. We used the L1 norm to constrain the left and right disparities to ensure continuous and smooth binocular disparity, as shown in formula 21:

$$L_{ds}^{l}=\frac{1}{N}\sum_{i,j}\left|\partial_{x}d_{ij}^{l}\right|e^{-\left\|\partial_{x}I_{ij}^{l}\right\|}+\left|\partial_{y}d_{ij}^{l}\right|e^{-\left\|\partial_{y}I_{ij}^{l}\right\|}$$ (21)

To sum up, the improved loss function is composed of the aforementioned three types of loss functions, as shown in formula 22:

$$L=\mu \left(L_{p}^{r}+L_{p}^{l}\right)+\lambda \left(L_{lr}^{l}+L_{rl}^{r}+L_{ds}^{l}+L_{ds}^{r}\right)$$ (22)

### 2.3 Evaluation method

In clinical applications, the doctor’s subjective evaluation is the most important factor in judging the image quality. There is no gold standard available for quantitative assessment especially in laparoscopic and endoscopic images (Zhang et al., 2022). Therefore, to verify the performance of tissue blood vessels, brightness, and color enhancement, we define two evaluation metrics: 1) Peak Signal-to-Noise Ratio (PSNR), and 2) Structural Similarity Index (SSIM).

PSNR and SSIM were used to evaluate image quality. PSNR is a measure of the quality of image reconstruction. The higher the PSNR value, the better the image quality will be. The formula is as follows:

$$MSE=\frac{1}{\text{mn}}\sum_{i=0}^{\text{m}-1}\sum_{j=0}^{n-1}{\left[I\left(i,j\right)-K\left(i,j\right)\right]}^{2}$$ (23)

$$PSNR=10\times \log_{10}\left(\frac{MAX_{I}^{2}}{MSE}\right)$$ (24)

where MSE represents the mean square error; $MAX_{i}^{2}$ represents the maximum possible pixel value of the image; I(i,j) represents the original image; and K(i,j) represents the noise image.

SSIM is used to measure the similarity of two images. The larger the SSIM value, the more similar the two images are. The formula is as follows:

SSIM(x,y)=[l(x,y)⋅c(x,y)⋅s(x,y)] (25)

$$l\left(x,y\right)=\frac{2\mu_{x}\mu_{y}+c_{1}}{\mu_{x}^{2}+\mu_{y}^{2}+c_{1}}$$ (26)

$$c\left(x,y\right)=\frac{2\sigma_{x}\sigma_{y}+c_{2}}{\sigma_{x}^{2}+\sigma_{y}^{2}+c_{2}}$$ (27)

$$s\left(x,y\right)=\frac{\sigma_{xy}+c_{3}}{\sigma_{x}\sigma_{y}+c_{3}}$$ (28)

where *μ* represents the mean, σ represents the variance; and $\sigma_{xy}$ represents the covariance of x and y ; $c_{1}={\left(k_{1}L\right)}^{2}$ and $c_{1}={\left(k_{2}L\right)}^{2}$ represent two constants, with $k_{1}=0.01$ and $k_{2}=0.03$ ; and L represents the range of image pixels.

## 3 Results and discussion

### 3.1 Data set and training parameter settings

Our experimental conditions are 64-bit Windows 10 operating system, using Intel(R) Core(TM) i7-10750H CPU; 32 GB RAM; NVIDIA 12 GB 3080Ti GPU. Install CUDA9.0 and use cuDNN7.0 for acceleration. On this basis, the U-Net model is built on the Tensorflow1.10.0 framework, as shown in Supplementary Table S1.

The dataset adopts the updated laparoscopic binocular dataset from the Hamlin Center (Chen et al., 2017). The left eye image is used for smoke cleaning. The binocular data are used for disparity estimation. The experimental dataset has a total of 34,240 pairs of binocular laparoscopic training images and 7,000 pairs of test images. This article divides the training images, of which 30,000 pairs of laparoscopic images are used as training sets and 4,240 pairs of validation images. Since many images in the laparoscopic dataset originally contain images of smoke, we perform supervised learning after fogging the images. The fog in the original image will affect the performance of the model, so we selected images that do not contain fog from the dataset to add fog. To ensure the reliability of the experimental data, each round of experiments is tested on synthetic smoke images and real smoke images. They were used in ten-fold cross-validation experiments. After training and validation separately, we used the test set to test, repeat this process ten times, and finally take the average of the ten results as the evaluation of algorithm accuracy. The synthetic image test set contained 1,000 images and the real smoke dataset contained 129 images. After fog rendering was performed on each image as a training set, the rendered images were divided into two levels: light fog and dense fog. During the training process, all images were first resized to a fixed size of 256 × 128, and then input to the model, the mean square error loss function was used, Adam is used as the optimization, the batch is set to 16, and the initial learning rate is set to 0.0001. The experiment adopts the control variable method, and conducts four sets of experiments for two levels of fog: including U-Net network, U-Net network plus CBAM attention mechanism, U-Net network plus Laplace transform, U-Net network plus CBAM attention mechanism, and Laplace transform.

The average training time of each model group is 4.5 h. According to the different levels of smoke and different model combinations, when the average loss is reduced to 0.02–0.03 in the light fog image training set, it will no longer decrease, and overfitting will not occur. The average loss on the validation set drops to around 0.3 and no longer decreases. When the average loss is reduced to 0.03–0.04 in the training set of dense fog images, there is no drop and no overfitting. The average loss on the validation set drops to around 0.4 and no longer drops. After training, export the model. This article can perform smoke purification on the synthetic image dataset. In order to apply it to engineering practice, this article uses the real dataset containing smoke; this model can purify real smoke.

### 3.2 Experiment results of dehazing of laparoscopic images

**FIGURE 7:**
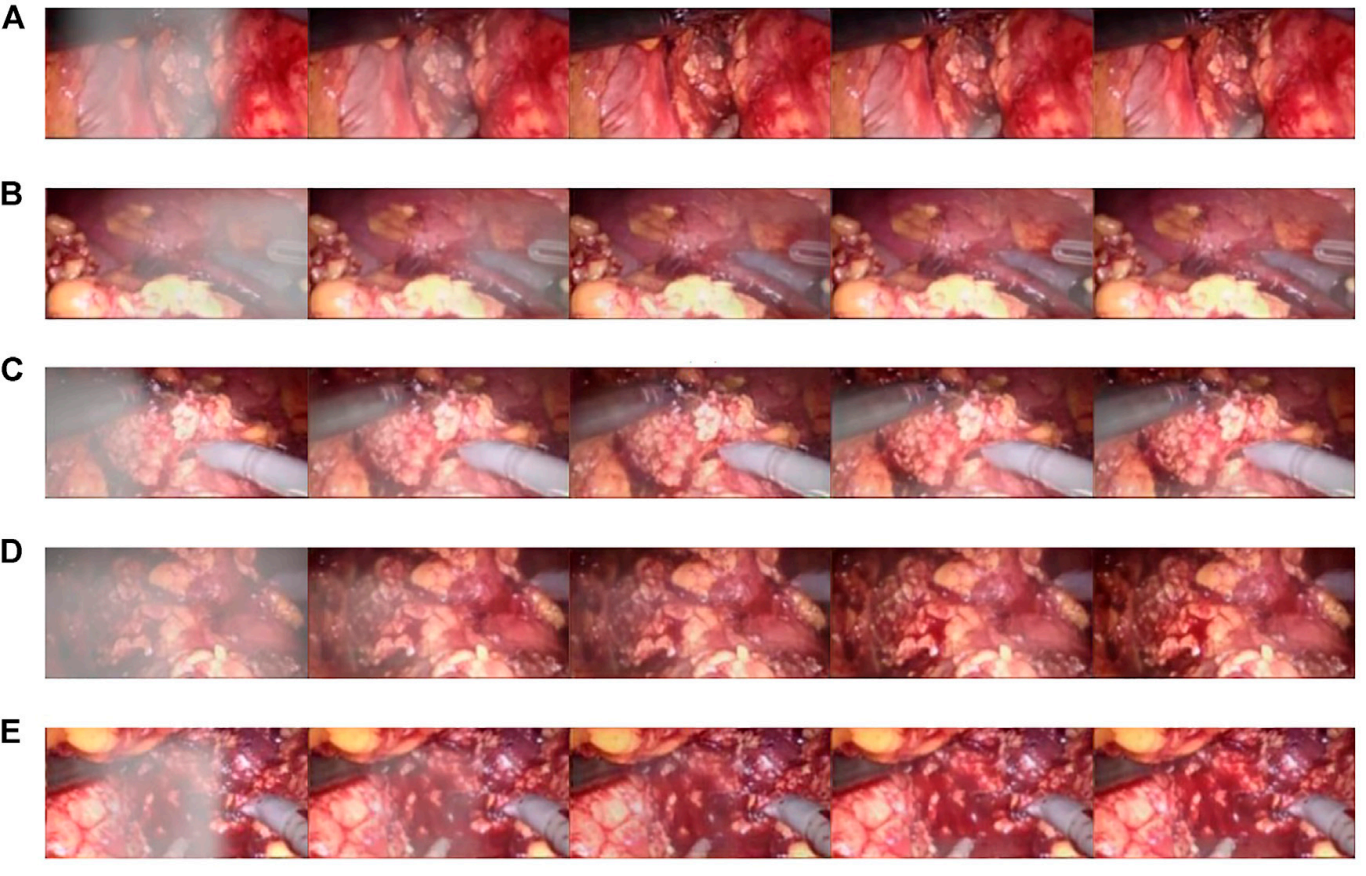
Synthetic smoke laparoscopic images and purified images. **(A)** Laparoscopic image with synthetic smoke and after purification; **(B)** Laparoscopic image with U-NET; **(C)** Laparoscopic image with U-NET + BAM; **(D)** Laparoscopic image with U-NET + Laplace; **(E)** Laparoscopic image with U-NET + CBAM + Laplace.

In addition, the test results of the synthetic dataset are shown in Figure 7. Figure 7A shows a synthetic smoke image, which is characterized by thick smoke and blocking of the original tissue structure. Figure 7B shows the results of using the original U-NET. There is still some residual smoke and the effect is not good. We used the Laplace pyramid transform to completely purify the smoke in Figure 7C. But, the brightness and color saturation of the bright parts of the original image were reduced. The smoke can be effectively purified with good color retention after the Laplacian pyramid transform is added in Figure 7D and Figure 7E.

In order to verify the validity of the model, we used the light fog dataset to conduct comparison experiments with other parameter settings under the same control experiment. The results are shown in Table 1. The training loss of adding the CBAM module alone is 0.023, and adding the Laplace transform alone is 0.038. In the case of Laplacian pyramid transform and the CBAM attention mechanism, the training loss of the model was 0.026. The CBAM module can better optimize the model. In terms of processing time, the CBAM module achieved a good result of 106.4pfs, and the best PSNR value was 31.435 dB. On the SSIM index, the experiment of adding the Laplacian pyramid obtained the best effect of 0.98.

**TABLE 1:** Model performance verification.

Model	PSNR	SSIM	PFS	Loss
U-Net	30.522	0.936	72.256	0.045
U-Net + CBAM	31.435	0.966	106.40	0.023
U-Net + Laplace	31.126	0.977	74.074	0.038
U- Net + CBAM + Laplace	31.045	0.980	90.191	0.026

### 3.3 Three-dimensional display experiment

Resnet50 was used for training; the training time was 7–8 h; the final loss obtained by training was 0.06. When HS-Resnet50 was used for training, the final loss was about 0.05. There was no overfitting in both schemes. The loss of HS-Resnet50 was lower, and the model training effect was better.

**FIGURE 8:**
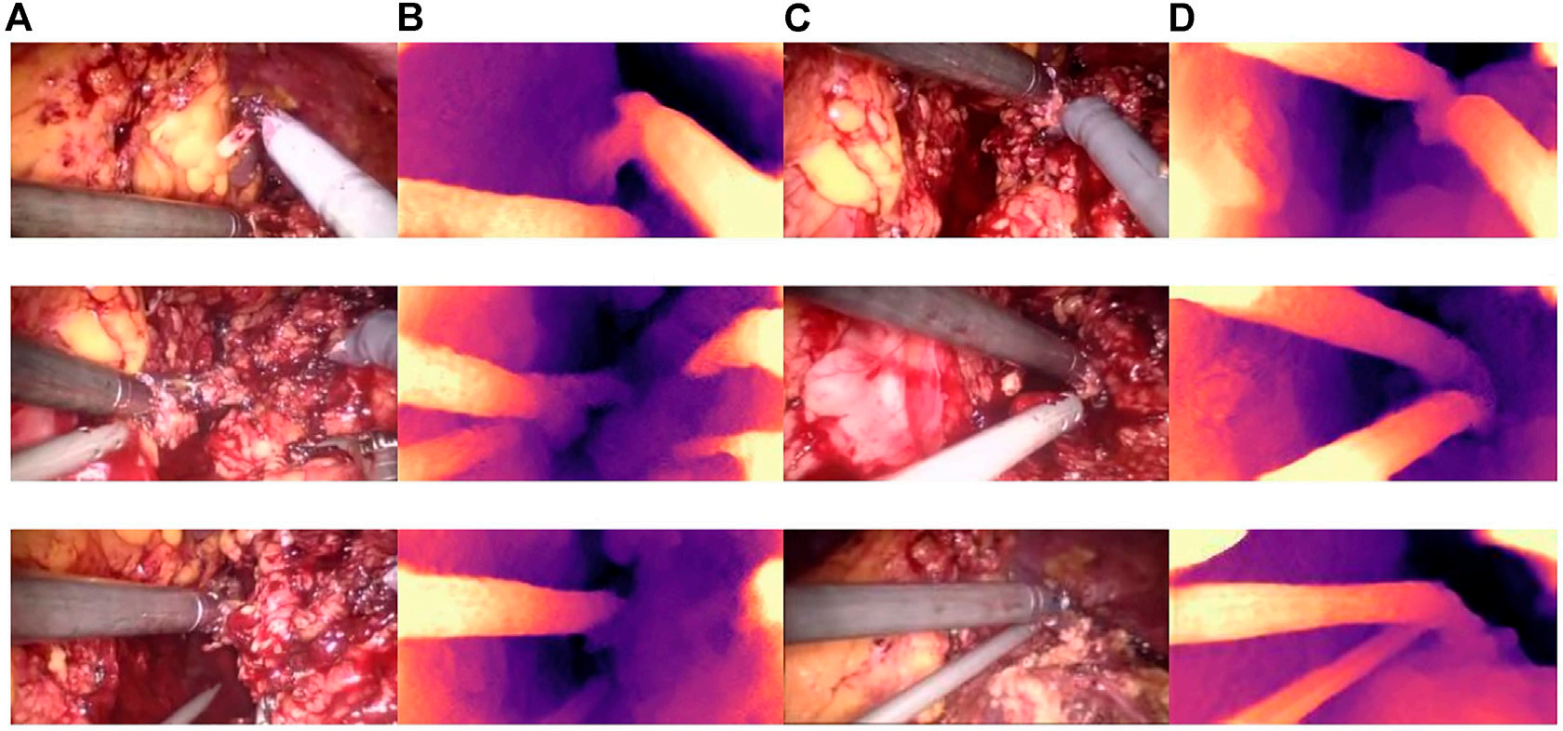
Parallax estimation results. **(A,C)**: endoscopic test images. **(B,D)**: the parallax image obtained using the HS-RESNET.

Qualitative test results are shown in Figure 8. Figure 8A and Figure 8C show the endoscope test images. Figure 8B and Figure 8D show the RGB parallax images obtained using the HS-Resnet model. It can be seen from the test images that the parallax images generated by the proposed model are complete and continuous, without any void phenomena. In the parallax images, a light-colored part is an object close to the camera and a dark-colored part is an object far from the camera. It can be confirmed from the original image that the distance relationship in the parallax images generated by this model is accurate.

**FIGURE 9:**
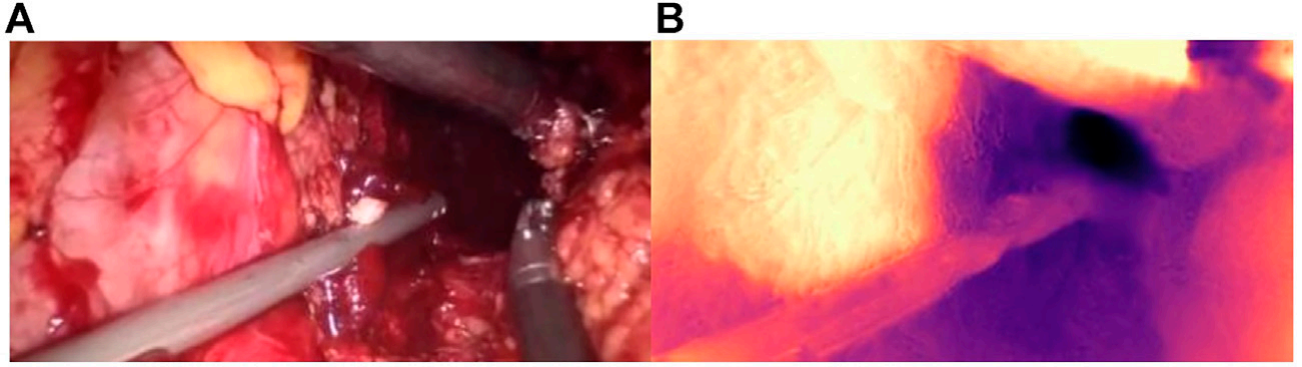
Details of the parallax estimation. **(A)**: Raw endoscope image; **(B)** proposed model parallax.

As shown in Figure 9, the binocular endoscope depth-estimation algorithm based on the improved HS-Resnet model can effectively obtain the disparity image while retaining the image details. The blood vessels in the abdominal cavity in Figure 9A are well preserved in the parallax Figure 9B. The original tissue texture of the image can be observed through the parallax image. The blood vessel information is very important in medical images, highlighting the blood vessels in the image and more. More details can also prevent doctors from accidentally injuring patients.

**FIGURE 10:**
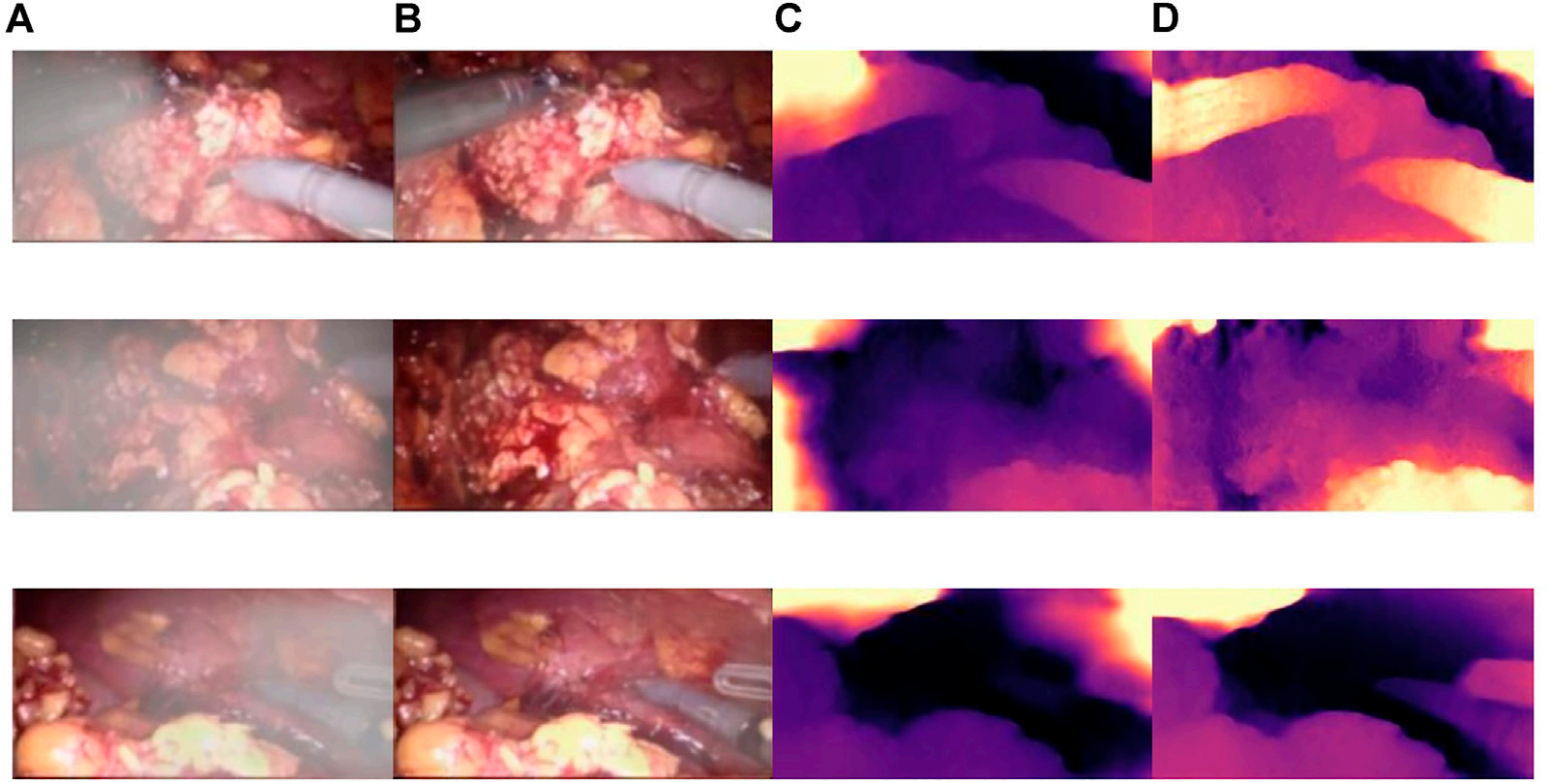
The effect of smoke on disparity estimation. **(A)** Synthetic smoke images; **(B)** smoke-removed images; **(C)** disparity images of the smoke containing images; **(D)** disparity images of the smoke-free images.

**FIGURE 11:**
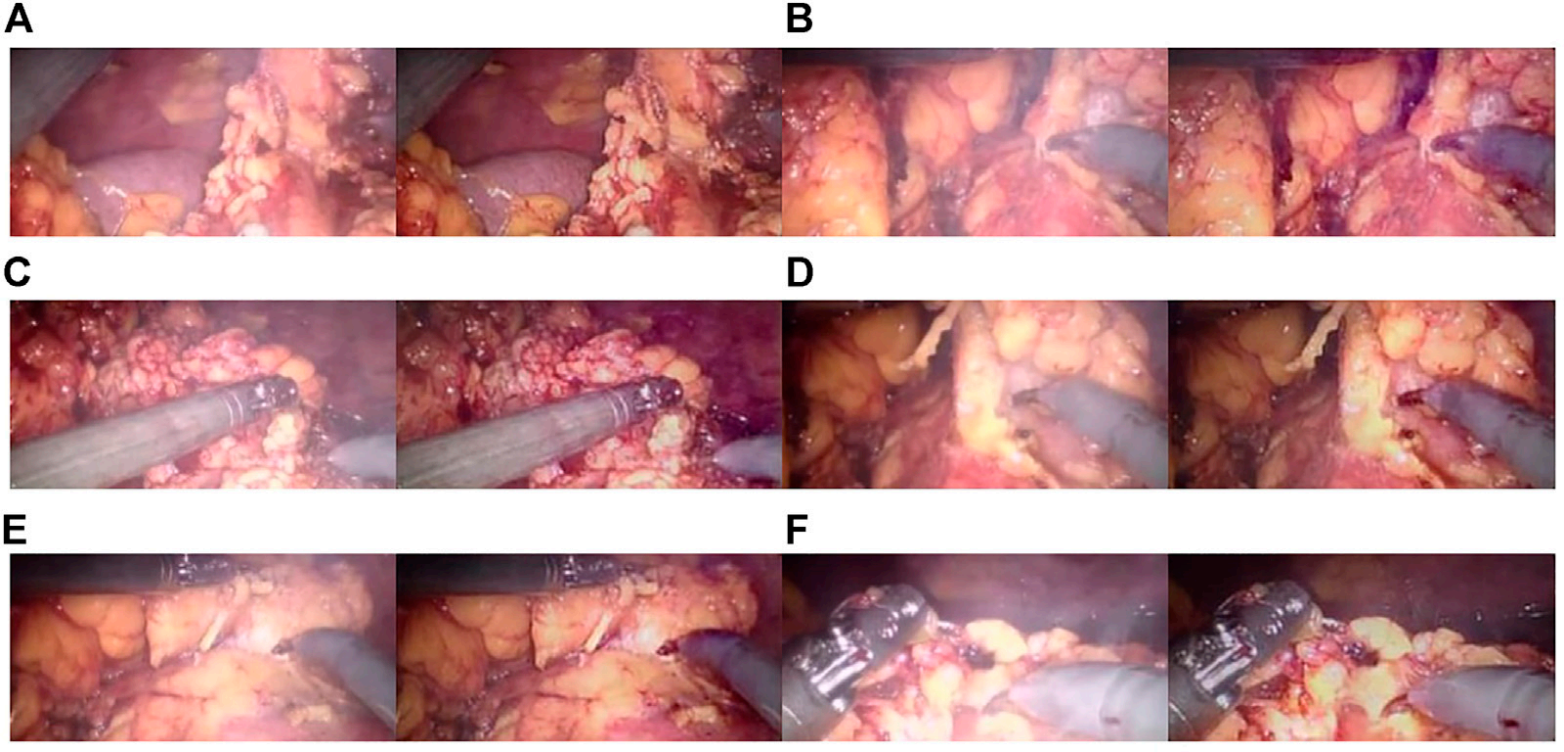
Laparoscopic images of real smoke and images after removal. **(A–F)** are randomly selected from the experimental results. Laparoscopic images of real smoke (left sub-panels) and images after smoke removal by U-Net + CBAM + Laplace pyramid fusion (right sub-panels).

Figure 10 shows the influence of smoke on disparity estimation. Figure 10A represents the synthetic smoke image, and it can be seen from the image that the smoke covers the front of the abdominal image. Figure 10B represents the image after chapter 3 smoke purification; it can be seen from the image that it no longer contains smoke. Figure 10C shows that the disparity value is obtained by performing a depth estimation on the image containing smoke. Due to the occlusion of the smoke, the disparity estimation is relatively blurred. The color is darker and it is difficult to distinguish the edge information. There are large black areas in the image that cannot be identified. Figure 11D shows the parallax estimation of the cleaned image compared with Figures 10C, D is lighter in color and easier to observe. The edge information image is clearer. The parallax estimation model in Figure 10C is occluded by smoke, which makes it difficult for the parallax estimation model to estimate the specific depth of human tissue. The parallax can be accurately estimated after the smoke is purified.

### 3.4 Smoke removal model performance verification

The CBAM attention module can effectively improve various indicators of the model. The Laplacian pyramid transform can better retain image details. The experimental results on real images are shown in Figure 11, and it can be seen from Figure 11A, Figure 11C, and Figure 11A that in surgery, real smoke generally blocks the doctor’s sight and fuzzes up the real vision in the scene. After removal, Figure 11B, Figure 11D, and Figure 11F show that the image processed using this model can purify smoke in the figure so that the fuzzy images are clearer.

In each subimage, the left is a smoke image randomly captured from a real surgery video and the right is the smoke removal result of the CBAM + Laplace image pyramid fusion + U-NET model.

The first image in each subimage is a smoke image randomly selected from a synthetic smoke dataset; the second image shows the smoke removal results of the basic U-NET model; the third image shows the smoke removal results of the CBAM + U-NET model; the fourth image shows the smoke removal results of the Laplacian image pyramid fusion + U-NET model; and the fifth image shows the smoke removal results of the CBAM + Laplacian image pyramid fusion + U-Net model.

**FIGURE 12:**
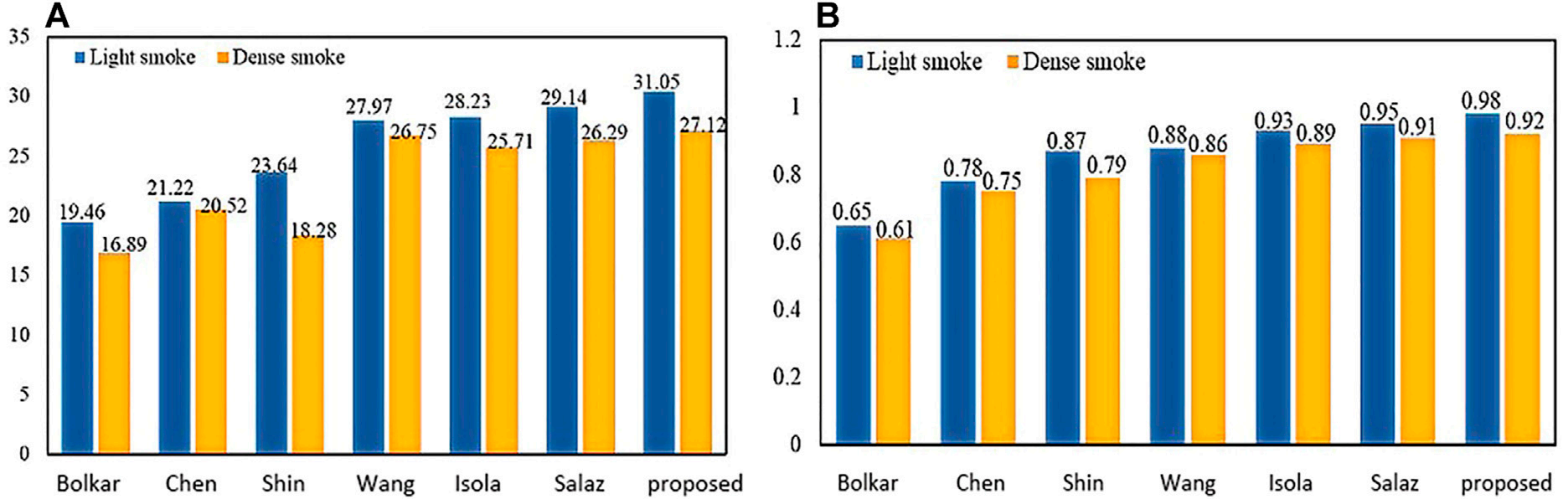
PSNR and SSIM comparison: **(A)** PSNR comparison; **(B)** SSIM comparison.

To verify the effectiveness of this model, we compared the frames per second (fps) of this article with six other methods. As shown in Table 2, our 90.19 (Fps) is inferior to GAN. But it achieves the best results on two important metrics (PSNR and SSIM) in Figure 12. The requirements for clinical endoscopic surgery have been met.

**TABLE 2:** Processing time comparison.

Methods	Model	Training images	Time (fps)	Platform
Bolkar et al.	CNN + DCP	Abdominal Cavity Images	32.40	Python(Caffe)
Chen et al.	CNN	Abdominal Cavity Images	89.14	Python(TensorFlow)
Shin et al.	physical method	Natural Images	1.28	Matlab
Wang et al.	U-Net	Abdominal Cavity Images	24.00	Python(Keras)
Isola et al.	GAN	Abdominal Cavity Images	120.0	Python(Pytorch)
Salazar et al.	GAN + DCP	Abdominal Cavity Images	92.19	Python(Pytorch)
Our Proposed method	U-Net + CBAM + Laplace	Abdominal Cavity Images	90.19	Python(TensorFlow)

Figure 12A shows the PSNR comparison between our method and the other six methods. Figure 12B shows the SSIM comparison between our method and the other six methods. Bolkar et al. (Bolkar et al., 2018) derived the atmospheric diffusion model and implemented it with a neural network. It is an earlier classic method in the field of smoke purification, so the results of various indicators are lower compared with recent methods. Chen et al. used a synthetic dataset. The U-Net architecture is used to achieve smoke purification. Among the several methods compared, the time performance is better. But the purification effect on real smoke images is poor. Shin et al. (Shin et al., 2019) adopted the radiation reflectance optimization scheme. The processing speed of a single image is the slowest. Wang et al. adopted the U-Net architecture and improved the down-sampling part. Compared with the first three methods, the PSNR index is greatly improved. Isola et al. (2017) used the adversarial neural network method, and achieved the best results in time performance. Salazar et al. (Salazar-Colores et al., 2020) used an adversarial neural network and took the dark channel-detected image as input and achieved good performance in various indicators. We used PSNR and SSIM in PSNR and SSIM. The two indicators have achieved the best results among several methods. In terms of time performance, the time indicator can achieve a stable display playback without jitter, so it can be applied in real-time systems.

### 3.5 Three-dimensional model performance verification

There are few literature studies on disparity estimation of endoscopic images. The evaluation indicators are not unique. Basic (Ye et al., 2017) used DeConvNet as the basis of the model network and adopted a self-supervised scheme. The disparity image obtained by training endoscopic images and the original image are used as the comparison standard, taking the structural similarity SSIM as the indicator. ELAS (Geiger et al., 2010) triangulated the matching points of the binocular image, making the surrounding points easier to match. SPS (Yamaguchi et al., 2014) proposed a new target optimization algorithm to solve the occlusion problem. The algorithm preserved the connectivity of image segments and utilized shape regularization in the form of boundary lengths. The algorithm finally realized image segmentation and disparity estimation for natural scene images. Siamese (Xu et al., 2019) is a stereo-automatic encoding and decoding structure, which is similar to monocular. The input codec structure is Basic. The initial disparity image is obtained from the codec structure. Then, the virtual view is obtained by the STN network. The loss is obtained by comparing the difference between the real view and the virtual view. One layer gets suitable parameters. Compared with the method proposed, the Siamese results obtained by binocular images are better than the Basic results obtained by monocular images. The SSIM effect reaches 0.726 ± 0.085, which is better than the Siamese results as shown in Table 3:

**TABLE 3:** SSIM comparison.

Model	Basic	ELAS	SPS	Siamese	Our proposed
Mean SSIM	0.555	0.473	0.547	0.604	0.726
Std SSIM	0.106	0.079	0.092	0.106	0.085

The parallax image obtained by SLAM is the true value. Using SSIM and PSNR as standards, we compared the predicted parallax value with the true value. The results are shown in Table 4. Our proposed average SSIM and PSNR results were 0.8826 ± 0.0678 and 17.2594 ± 1.6254, respectively. The results showed that the proposed method is superior to other methods.

**TABLE 4:** PSNR and SSIM comparison.

Model	Basic	Autoencoder	Our proposed
Mean SSIM	0.5414 ± 0.0709	0.8349 ± 0.0523	0.8826 ± 0.0678
Mean PSNR	7.7650 ± 1.3686	14.4957 ± 1.9676	17.2594 ± 1.6254

The experiments use the binocular heart data in the Hamlin endoscopy dataset. This dataset originally did not contain ground truth disparity values. Several algorithms are compared in Table 5. Godard et al. obtained the disparity image by extracting image features through CNN in the natural scene dataset. The parallax information from the left image to the right image is imaged to obtain the virtual view. The loss value is obtained by comparing the virtual view with the real view. The model results obtained from this training perform well on natural scene datasets. Wang et al. (Wang et al., 2018b) used variational disparity estimation technology to minimize the global energy function of the entire image. Based on the grayscale and gradient constants, they supposed that a data term and a local and non-local smoothing term were defined to construct the cost function. The real disparity image was obtained. Stoyanov et al. (Stoyanov et al., 2010) and Luo et al. (Luo et al., 2019) used two encoders and decoders to extract the disparity images for the left and right images, respectively. They used the traditional binocular algorithm AD-CENSUS to generate unsupervised training. The surrogate disparity labels, which guide the training process, achieved better results than the previous two literature studies on both MAE and RMSE metrics. This article compares the results with the aforementioned four methods. From the experimental results, we find that our result has a certain improvement in MAE. The RMSE index has a larger improvement than the aforementioned methods.

**TABLE 5:** MAE and RMSE comparison.

Model	Methods	MAE, mm	RMSE, mm
Heart 1	Godard et al	2.39 ± 0.62	2.99 ± 0.61
	Wang et al	2.16 ± 0.65	-
	Stoyanov et al	2.36 ± 0.92	3.88 ± 0.87
	Luo et al	1.84 ± 0.40	2.69 ± 0.58
	Our Proposed	1.65 ± 0.35	2.45 ± 0.52
Heart 2	Godard et al	1.79 ± 0.40	2.65 ± 0.28
	Wang et al	2.14 ± 0.83	-
	Stoyanov et al	3.20 ± 1.15	4.85 ± 1.82
	Luo et al	1.49 ± 0.41	1.90 ± 0.38
	Our Proposed	1.45 ± 0.40	1.62 ± 0.42

In endoscopic image evaluation, the doctor’s subjective evaluation is still the important method to verify the image quality. The establishment of the quantitative assessment is a challenging task since there are no available gold standards. More specialized evaluations are needed to validate the effectiveness of 3D reconstruction methods for endoscopic images. Therefore, we invited 10 chief physicians from the Affiliated Hospital of Southwest Medical University with more than 5 years of laparoscopic surgery experience to score the 3D images. The subjective evaluation criteria referring to the Double Stimulus Continuous Scale (DSCQS), 3D effect, viewing comfort, and acceptability were rated on a scale of 1 (worst) to 5 (best). A score of 1 indicated a non-diagnostic image, and a score of 5 indicated an excellent diagnostic image quality. Pathological invariance was scored with 0 (change) or 1 (no change).

The evaluation of the 10 clinicians is shown in Table 6; our method received the best subjective quality evaluation. 3D effect, viewing comfort, and acceptability are better than other methods, and the improved loss function can better retain the details of medical images. It is proved that the proposed 3D reconstruction algorithm can be applied to clinical scenarios.

**TABLE 6:** Subjective evaluation and comparison of the 3D reconstruction effect. (Mean ± STANDARD, deviation).

Methods	3D Effect	Viewing comfort	Invariance	Acceptability
Godard[27]	3.8 ± 0.48	3.5 ± 0.11	0.3 ± 0.15	3.2 ± 0.21
Wang[40]	3.7 ± 0.72	3.6 ± 0.82	0.3 ± 0.55	3.5 ± 0.74
Stoyanov[41]	3.9 ± 0.91	3.8 ± 0.25	0.4 ± 0.51	3.8 ± 0.11
Luo[42]	4.1 ± 0.40	4.0 ± 0.35	0.6 ± 0.51	4.1 ± 0.63
Proposed	4.1 ± 0.69	4.1 ± 0.11	0.7 ± 0.12	4.2 ± 0.38

In this article, a total of 1,200 endoscopic images in the dataset were processed. The average processing time per image is 0.0275 s and 36fps was obtained. Therefore, it can meet the real-time requirements of a 3D display system.

**FIGURE 13:**
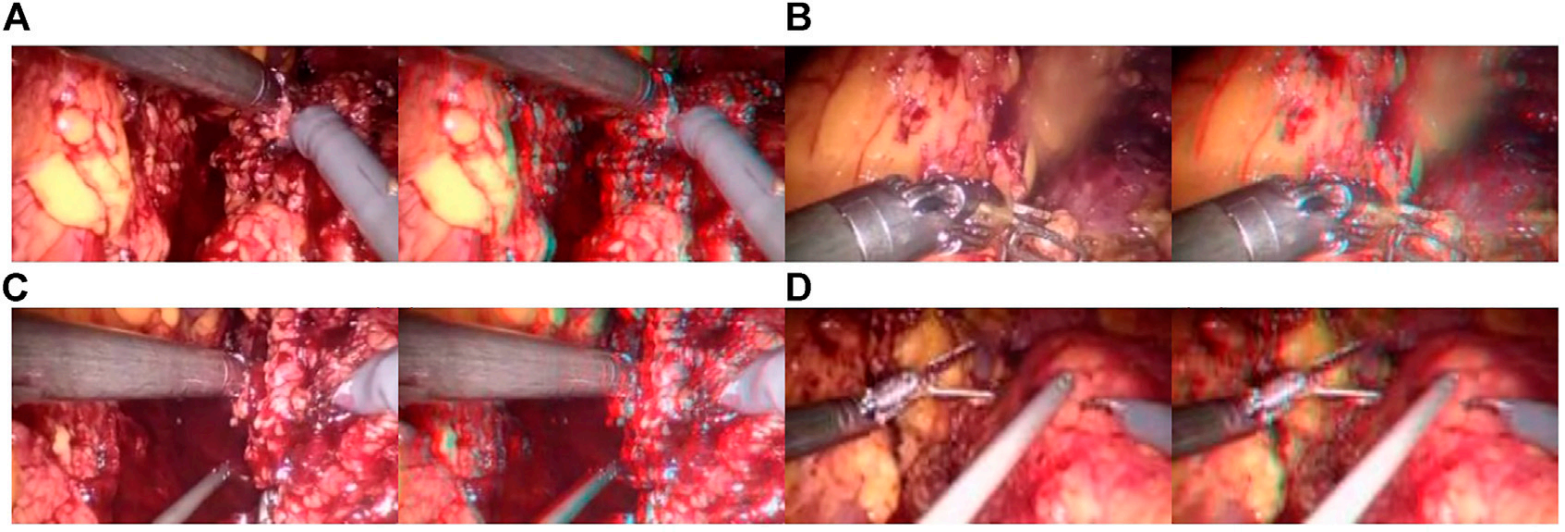
Red and blue 3D display images. The left image in each sub-image is the original view, and the right image is the chromatic 3D display image. **(A–D)** are randomly selected from the experimental results.

The improved U-NET network applied to an original endoscopic image can obtain a better parallax image with higher accuracy to obtain a better three-dimensional display effect. Moreover, void-filling and reverse imaging can be performed on the parallax image to recover a better right view, color offset can be performed on the left and right views, and the 3D display effect can be seen by wearing red and blue lenses, as shown in Figure 13.

**FIGURE 14:**
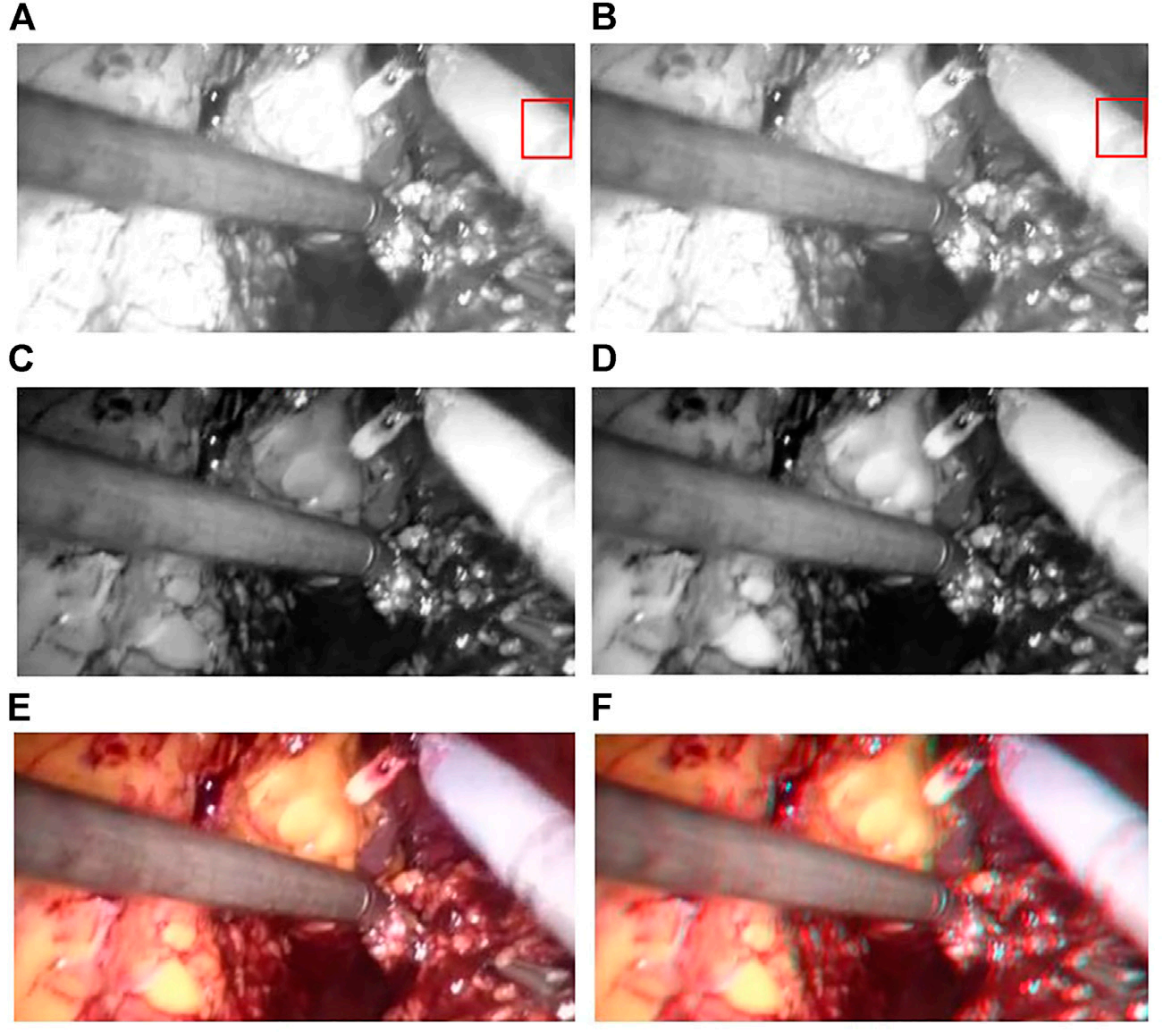
3D display of color difference; **(A)** Red Component; **(B)** Offset Red Component; **(C)** Blue Component; **(D)** Green Component; **(E)** Raw endoscope image; **(F)** 3D Display.

We validated the effectiveness of our method on the binocular laparoscopy dataset. For any image on the binocular laparoscopic dataset, an adaptive neural network endoscopic three-dimensional reconstruction method is proposed. If there is smoke, first use the smoke purification algorithm to obtain the purified image, and secondly obtain the disparity image. The result of 3D display is shown in Figure 14. Figure 14A represents the red component of the original image. Figure 14B represents the red component after fusion of parallax. There is a slight difference between Figure 14A and Figure 14B. It is difficult to observe with the naked eye. We need to carefully observe the slight difference between the red boxes on the right side of the image. There are certain wrinkles in Figure 14B. It shows that the red component has moved after parallax stacking. Figure 14B is a virtual image from another viewpoint. Figure 14C and Figure 14D represent the blue and green components separated from the original image, respectively, and Figure 14E represents the original image. The RGB images are shown in Figure 14A, Figure 14C, and Figure 14D, respectively. Figure 14F represents the color-difference three-dimensional display image. From Figure 14, we can find that the red–blue parallax movement range becomes larger, which is more suitable for human eye observation.

## 4 Conclusion

To meet the practical application requirements of binocular endoscopic medical images, this article organically combines a global expansion with a local adaptive expansion of the network structure. Aiming at the lack of real parallax in unsupervised binocular endoscopic images, we proposed a 3D reconstruction scheme for adaptively processing the smoke images. Subjective evaluation and objective evaluation were used for verification. The 3D effects in the subjective evaluation obtained an optimal value of 4.2 ± 0.38. In the de-hazing tests on real datasets, our method achieved an SSIM of 0.980, a PSNR of 31.545 dB, an average running speed of 90.191 fps, and a much lower training time than similar methods. The proposed self-supervised disparity estimation method also outperformed the existing methods, with an SSIM of 0.726 ± 0.085 and a PSNR of 17.2594 ± 1.6254 dB; MAE 1.45 ± 0.40, RMSE 1.62 ± 0.42. It meets the needs of medical images in various indicators and solves the real-time problem of clinical operations. The present article can therefore guide the development of endoscopy devices.

## Data Availability

The original contributions presented in the study are included in the article/Supplementary Material; further inquiries can be directed to the corresponding authors.
