# Supplementary material for: A 3D reconstruction based on an unsupervised domain adaptive for binocular endoscopy
Source: Front Physiol. 2022 Sep 1;13:994343. doi: 10.3389/fphys.2022.994343 (PMC9475117; doi:10.3389/fphys.2022.994343)
Supplement: Supplementary file 1 [file Table1.docx]

Table 1 Experimental configuration and network parameter setting

| Type | Configure | Network parameters | Setting |
| --- | --- | --- | --- |
| CPU | 2.6 GHz Intel i7 | Batch size | 16 |
| GPU | GTX 3080Ti GPU | Initial learning rate | 0.0001 |
| Graphics Memory | 16G | Epoch | 100 |
| Memory | 32G | Dropout | 0.5 |
| OS | Windows 10 | Iteration | 5000 |
| Framework | PyTorch1.0 | Optimizer | Adam |
