# Supplementary figures and images for: A 3D reconstruction based on an unsupervised domain adaptive for binocular endoscopy
Source: Front Physiol. 2022 Sep 1;13:994343. doi: 10.3389/fphys.2022.994343 (PMC9475117; doi:10.3389/fphys.2022.994343)

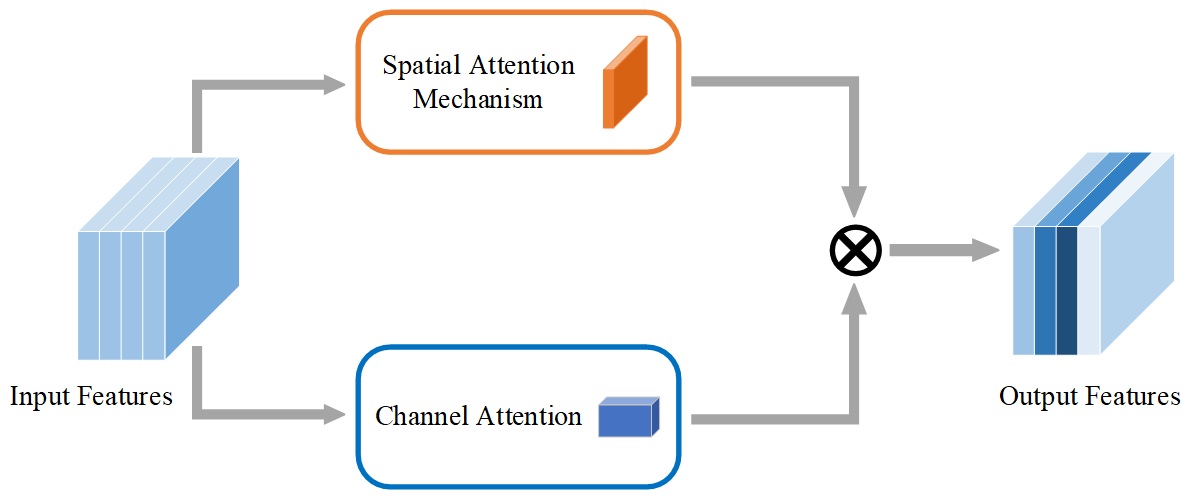

Supplement: Supplementary file 2 [file Image1.jpeg]
